# Atomic scale memristive photon source

**DOI:** 10.1038/s41377-022-00766-z

**Published:** 2022-03-29

**Authors:** Bojun Cheng, Till Zellweger, Konstantin Malchow, Xinzhi Zhang, Mila Lewerenz, Elias Passerini, Jan Aeschlimann, Ueli Koch, Mathieu Luisier, Alexandros Emboras, Alexandre Bouhelier, Juerg Leuthold

**Affiliations:** 1 ETH Zurich, Institute of Electromagnetic Fields, Zurich, 8092 Switzerland; 2 Laboratoire Interdisciplinaire Carnot de Bourgogne, UMR 6303 CNRS, Université de Bourgogne Franche-Comté, Dijon, 21078 France; 3 ETH Zurich, Integrated Systems Laboratory, Zurich, 8092 Switzerland

**Keywords:** Lasers, LEDs and light sources, Nanophotonics and plasmonics

## Abstract

Memristive devices are an emerging new type of devices operating at the scale of a few or even single atoms. They are currently used as storage elements and are investigated for performing in-memory and neuromorphic computing. Amongst these devices, Ag/amorphous-SiO_x_/Pt memristors are among the most studied systems, with the electrically induced filament growth and dynamics being thoroughly investigated both theoretically and experimentally. In this paper, we report the observation of a novel feature in these devices: The appearance of new photoluminescent centers in SiO_x_ upon memristive switching, and photon emission correlated with the conductance changes. This observation might pave the way towards an intrinsically memristive atomic scale light source with applications in neural networks, optical interconnects, and quantum communication.

## Abstract (web summary)

*Photon* emission is observed during the resistive switching process of memristors with an atomic-sized footprint and a scalable fabrication procedure.

## Graphical abstract

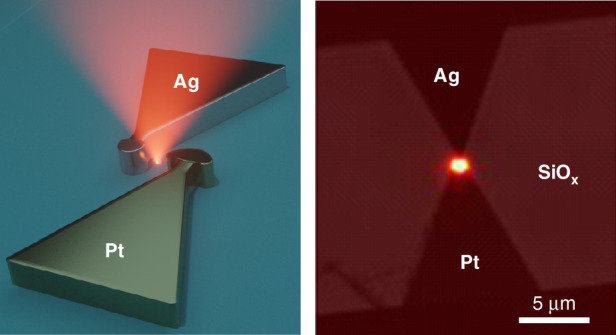

## Introduction

Compact on-chip photon sources are of great interest to the scientific community. Ideally, such light sources offer a compact footprint, low power consumption, are operated electrically and are compatible with the standard CMOS fabrication process, leading to high integration densities and energy-efficient operation at reduced cost^1^. Such compact electrically-driven photon sources would be much needed within integrated circuits. For instance, for optically interconnecting processor and memories^2^; or to optically communicate a sensing event^3^. In quantum communications, they could act as on-chip single photon sources. In the field of neuromorphic computing, they could be used to communicate a memristive state^4^.

Research in compact electrically-driven photon sources has led to quite a few innovative solutions over the past years. For example, quantum dot based light sources already deliver excellent emission efficiency with controlled spectra^5–7^ but require complex integration processes for fabrication. Electrically-driven light emitting tunnel junctions can be extremely compact and versatile^8–11^. However, the vertically stacked architectures still require a large injection area^8^ and fine control over the fabrication of a thin oxide barrier, whereas the in-plane architectures require advanced nanofabrication^9^ or stochastic arrangement^10^ that are not scalable.

In parallel, innovative atomic scale electronic devices have emerged notably with the advent of memristors^12^. Memristive devices are attractive for downscaling, as the operation only relies on the movement of a few atoms^13–15^. The low energy, high-speed operation^16^ makes memristors suitable for high-density storage^17^, in-memory computing, and neuromorphic computing^18^. Interestingly, these devices may be advantageously merged with optical functions: memristively controlled optical switches^4^ and photodetectors^19^ have been introduced. Yet, so far, the photonic operation of a memristor relies on external or co-integrated photon sources^20^.

In this paper, we introduce an atomic scale memristive device capable of emitting photons during resistive switching, superseding thus the need for an external optical source. Our device features the compact footprint of transistors and compatibility with the emerging memristive technology. The device is based on transient-mode electroluminescence (EL) triggered by memristive switching. More precisely, near-infrared emission occurs within the gap of an in-plane Ag/amorphous-SiO_x_ (a-SiO_x_)/Pt memristive junction when the resistance state changes. To enhance the emission, the apex of the Ag and Pt triangular electrodes are engineered to form a plasmonic nanoantenna. Our demonstration could potentially trigger a new conceptual paradigm for devices operating at the atomic level as electrical and optical functionalities may be embedded on the same nanoscale component. The paper thus addresses the challenge of downscaling photon sources similar to what we currently witness within electronics. Moreover, the new emitters offer not just light emission but are functional electrical devices on their own.

## Results

### Sample description and working principle

**Fig. 1:**
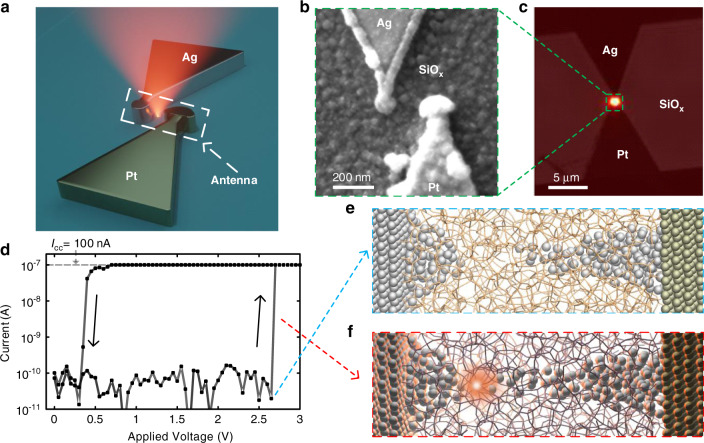
Overview of an atomic photon source (APS). **a** Schematic illustration of the atomic scale memristive photon source (APS) with the plasmonic nanoantenna (highlighted in white dashed box) forming the tips of the quasi-triangular-shaped Ag and Pt electrodes. **b** SEM image of one APS. The nanoantennas can be seen at the apex of the Ag and Pt electrodes. A layer of 60 nm sputtered SiO_x_ covers the chip and serves as the switching matrix. **c** Overlay of a wide-field electroluminescence image with an optical transmission image from the APS. The electroluminescence image of the APS is taken accompanying a resistive switching *I*–*V* voltage sweep. The bright spot signals photons emitted from the gap. The two images are overlaid to distinguish the configuration of the electrodes (darker areas). **d** A typical *I*–*V* curve for a voltage sweep of the APS, demonstrating a hysteresis behavior characteristic of a memristor. The photon emission is associated with the abrupt change of current, highlighted by the black arrows. Upon switching to the low resistance state, a compliance current of *I*_cc_ = 100 nA limits the maximum current flowing through the device. **e** Schematic illustration of the APS at the atomic level at the beginning of filament formation and device switching into the low resistance state. The grey spheres illustrate the Ag atoms constituting the active electrode and the filament. The green spheres represent the inert Pt electrode. The orange matrix mimics the a-SiO_x_ lattice. The Ag protrusions from both electrodes result from repeated Ag filament formation and dissolution from resistive switching cycles. **f** Schematic illustration of the atomic photon source emitting photons during the memristive switching process. The Ag filament is growing from the Pt towards the Ag electrode. The bright red spot represents the emitted photons at a likely origin in the gap of the atomic photon source

The atomic scale memristor photon source presented here, referred to as atomic photon source (APS), consists of an Ag/a-SiO_x_/Pt memristive switching in-plane junction fabricated on a glass coverslip. For more information on the fabrication process, the readers are referred to Supplementary Section I. An APS’s conceptual illustration and a scanning electron microscope (SEM) image are shown in Fig. 1a and b, respectively. By applying a voltage between the Ag and Pt electrode, resistive switching is achieved by the formation and dissolution of a conductive Ag filament^13,15^. We discover that light is emitted within the gap between the Ag and Pt electrode during these critical forming and dissolution phases. To enhance the radiation and collection efficiencies, a plasmonic nanoantenna is connected to the apex of the two quasi-triangular metal contact electrodes. The number of photons emitted by a single device is sufficient for detection with a standard CCD sensor, as shown in Fig. 1c (the setup is described in Fig. S1 of the Supplementary). Here an optical transmission image of one APS is overlaid with the photons detected during its operation (see Fig. S2 in the Supplementary for the two separate optical images).

A switching cycle of the APS is shown in Fig. 1d. It is characteristic for a memristive threshold switching device^21,22^. Once the switching voltage of ~2.6 V is reached during the voltage ramping up, the device rapidly switches from a high resistance state to a low resistance state and reaches a set compliance current of *I*_cc_ = 100 nA. The low resistance state is volatile; as a result, the device will switch back to the initial high resistance state below a certain voltage threshold, which is around 0.3 V in this example. The release of photons is observed during the abrupt current change (highlighted by the black arrows), as illustrated in Fig. 1e and f. We attribute the transient-mode photon emission mechanism to luminescent defects—oxygen vacancy clusters—generated during resistive switching and thus filament growth and dissolution, which will be discussed below in more detail.

### Investigation of electroluminescence

**Fig. 2:**
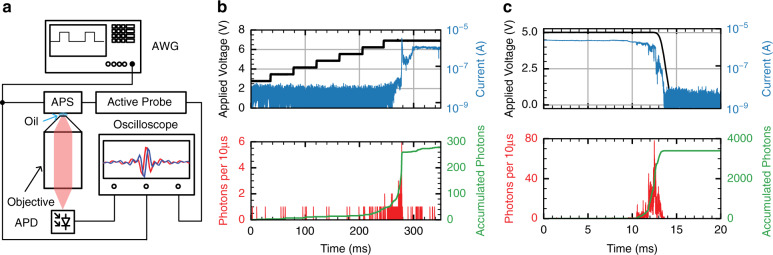
APS Electroluminescence Measurement. **a** The setup of the measurement. The APS sample is placed on top of an inverted microscope and is imaged by using an oil immersion objective. An avalanche photodiode records the optical activity of the device. A voltage bias is applied to the device under test (APS) by an arbitrary wavefunction generator (AWG). The current and the emitted light are measured synchronously by an oscilloscope. **b** Upper panel: current evolution upon filament formation. The applied voltage is gradually ramped up to 7 V, shown in the black curve. At ~280 ms, the current increases abruptly from the noise floor of ~10 nA to several μA, shown in the blue curve. Lower panel: corresponding photon activity. A burst of photons can be seen upon the abrupt increase of current. Both the photon rate and accumulated photon counts are plotted. Red curve: binned photon counts with a size of 10 μs. Green curve: accumulated photon counts. Most photons are generated within the transient switching time at around 280 ms. **c** Emission from filament dissolution, a burst of photons can be seen upon the abrupt current drop at around 12 ms. Photon counts are binned every 10 μs

The transient-mode photon emission and its relations to the applied voltage and current are investigated by time-resolved electroluminescence (EL) measurements. The EL measurement setup is illustrated in Fig. 2a. The sample containing the device under investigation (APS) is placed on top of an inverted microscope and is imaged by using a 100X oil immersion objective with a numerical aperture of N.A. = 1.49. The emitted light, collected through the glass substrate, is then directed to an avalanche photodiode (APD) and is counted synchronously with the electrical signals. The APS is electrically driven by an arbitrary waveform generator (AWG), and the signals are measured by an oscilloscope. In Fig. 2b, an exemplary measurement is depicted, where the voltage is gradually increased (shown in black in the upper panel). Between 250 and 280 ms, the current rises from the noise floor of ~10 nA to several μA (shown in blue in the upper panel), signaling the formation of a filament. The photon counts (shown in red in the lower panel) and accumulated photon counts (shown in green in the lower panel) clearly show that a burst of light is generated during the resistive switching. Upon turning off the voltage (Fig. 2c), photons are again generated while the filament is dissolved, as indicated by the concomitant current drop. The transient photon emission is distinctively different from what is observed in a tunnel junction emitter, where the inelastic electron tunneling leads to a continuous emission for a constant resistive state^10^. The difference of transient photon emission and inelastic electron tunneling is discussed in more detail in the Supplementary Section X.

**Fig. 3:**
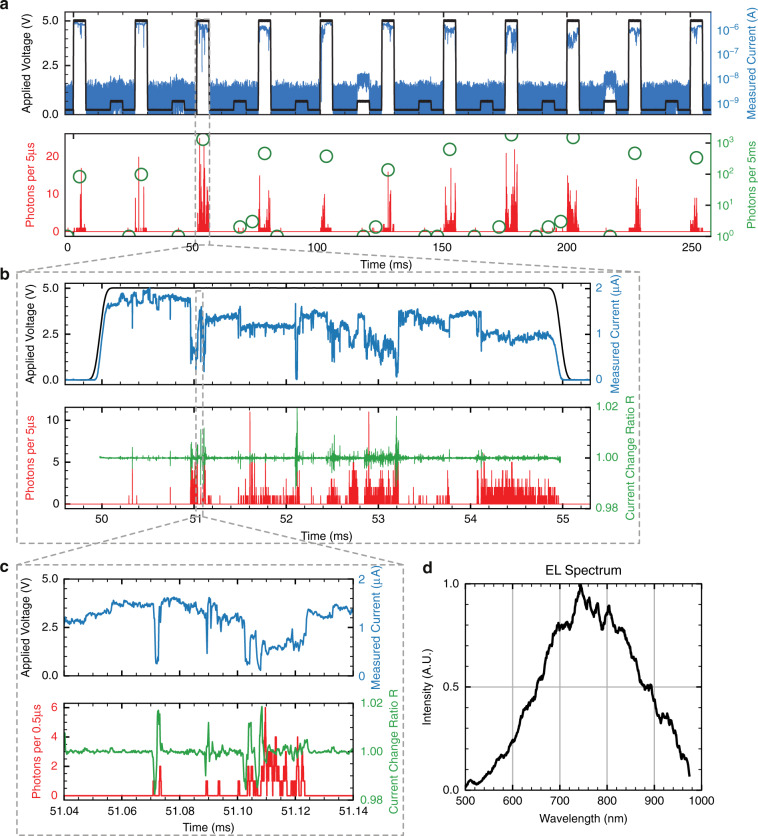
APS Electroluminescence Pulse Measurement. **a** Upper panel: A series of alternating 5 V, 5 ms and 0.5 V, 5 ms pulses is applied to the APS. The measured current is shown on the right axis. Lower panel: Corresponding photon counts. Left axis (black bars), photon counts are binned every 5 µs. Right axis (green circles), photon counts are binned every 5 ms. **b** Upper panel: zoomed-in plot of one of the pulses (encircled in grey) from a. Lower panel left axis: photon counts are binned every 1 µs. Lower panel right axis: the current change ratio ***R*** ***=I*** _**t**_ ⁄ ***I***_**t-∆t**_. A moving average window of 1 µs is applied to ***R*** to reduce the noise. **c** Zoomed-in plot of b. Photon counts are binned every 0.5 µs. A moving average window of 1 µs is applied to *R* to reduce the noise. **d** Measured EL spectrum with a series of 5 V, 5 ms pulses. The QE of the whole optical path including the spectrometer has been compensated

Despite atomic dimensions, the APS features a robust operation, as depicted in Fig. 3a. Here, a series of alternating 5 V and 0.5 V voltage pulses of 5 ms duration are applied to the device. The amplitude of the 0.5 V “read” pulses is not high enough to cause a growth of the filament and is used to demonstrate that the device stays in the low resistance state (LRS) after the 5 V pulses. The upper panel displays voltage and current, and the lower panel in Fig. 3a shows the simultaneously acquired photon activity displayed for two binning times. Clearly, the device emits bursts of photons whenever an electrical stress is applied. It should be noted that unlike the previous experiments in Fig. 1d and Fig. 2, no compliance current has been applied here. This mode of operation with a relatively high voltage (5 V) allows for repeated changes of the filament’s structure caused by electrical stress. More specifically, the relatively high voltage applied across the gap enables repeated Ag filament formation. Once the filament is formed, the transport of charges destabilizes the filament structure via local Joule heating^15^, and a renewed growth of the filament can take place. These repeating cycles driven by the motion of a few atoms during a voltage pulse lead to a current fluctuation. This current fluctuation can be seen more clearly in the zoom-in plot of a single voltage pulse in Fig. 3b and c.

To correlate the optical activity with the change of current, we define the ratio of the current variation as *R* *=* *I*_t_ ⁄ *I*_t–∆t_, which represents the rate of filament structural changes. A large |*R*| signals the morphological atomic reorganization of the conductive filament within the switching layer. The ratio is displayed in the bottom frame of Fig. 3b and Fig. 3c (green curve), together with the number of photons detected during the pulse (red curve). A correlation between the photon counts detected and *R* can be readily observed: the photon counts are high whenever |*R*| is large. There is barely any photon emission when *R* is close to 0. This is even clearer in the zoomed-in plot of the pulse shown in Fig. 3c, as highlighted by the grey dashed box. From the correlation of *R* and the photon counts, we conclude that such an electrically-induced structural change is systematically correlated with the emission and ultimately the cause for releasing the photons.

The APS presented here has been tested for ~100 cycles during which photon emission (electroluminescence) has been observed. It is worth noting that in each cycle the device is going through many filament formation and dissolution cycles even within a single electrical pulse excitation, therefore the photonic-emission (optical) endurance cannot be directly compared with the electrical endurance. Still, electrical endurance is one of the most critical measures of memristive devices^21^, and electrical endurance tests have previously been performed on devices with a similar material stack^16^. Those devices performed 50,000 consecutive cycles without a single failure.

The spectrum of the emitted photons taken during a series of 5 V, 5 ms pulses is shown in Fig. 3d. The APS features a broad emission spectrum spanning the near-infrared region with an emission peak at ~745 nm. The spectrum helps to identify the emission mechanism, as argued later in the Discussion section.

### Emission efficiency optimization

To optimize the quantum efficiency of the APS, a plasmonic nanoantenna at the tip of the APS electrode has been designed to engineer the electromagnetic environment. To that aim, we constructed a geometry tuned to the emission peak shown in Fig. 3d. The antenna dimensions have been optimized to maximize the number of photons collected by the objective beneath the sample. This is done by maximizing the radiation efficiency (*η*_*z*-_), the fraction of photons emitted towards the *z*- half-space.

**Fig. 4:**
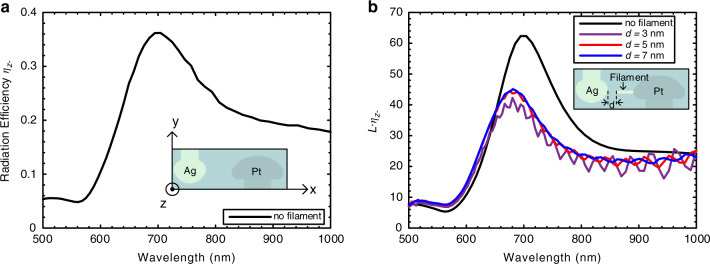
Investigation of the APS and the influence of the antenna and the filament on its emission. **a** Simulation of the antenna radiation efficiency (effect of filament not included). Inset, the Ag and Pt nanoantenna structure considered for the simulation are shown together with a definition of the orientation. The shape of the antenna is extracted from the SEM image shown in Fig. 1. **b** Simulated quantum efficiency *L* · η_z-_ as a function of the filament-antenna gap *d*. Inset, the structure of the Ag and Pt nanoantenna used in the simulation. The filament is modeled as a cylindrical Ag rod with a radius of 0.5 nm

The simulated *η*_*z*-_ of the fabricated APS is depicted in Fig. 4a. The inset shows the geometrical structure model. The simulation confirms a resonance peak at ~710 nm with a high radiation efficiency maximum of ~36% as well as an efficiency above 20% for a broad spectral range of 630 to 860 nm.

In addition, simulations with an Ag filament between the two electrodes are performed to estimate its effect on the optical quantum efficiency of the APS (details see Supplementary Section IV). The quantum efficiency of the APS is approximately proportional to the product of *η*_*z*-_ and the local density of optical states (LDOS) enhancement *L* (details see Supplementary). In Fig. 4b, this factor *L* ∙ *η*_z-_ is investigated for different gap sizes between the filament and the silver antenna. As shown by the simulations, *L* ∙ *η*_z-_ only drops slightly with the presence of a filament. This point is discussed in greater detail in the Supplementary Section IV. We conclude that engineering the extremity of the electrodes is a necessary route to further improve the efficiency of the APS.

### Investigation of the luminescence mechanism

To investigate the origin of the photon emission, a two-step analysis is conducted. Our hypothesis is based upon the creation of optically-active defect centers induced by the structural changes from resistive switching. To test this assumption, we perform photoluminescent (PL) measurements to investigate the presence of new species located in the gap after electrically stressing the APS. Second, various PL and EL measurements are performed in the presence and absence of silver, indicating that the luminescent centers likely originate from defects in the SiO_x_ matrix rather than from the silver.

### Investigation with photoluminescence

**Fig. 5:**
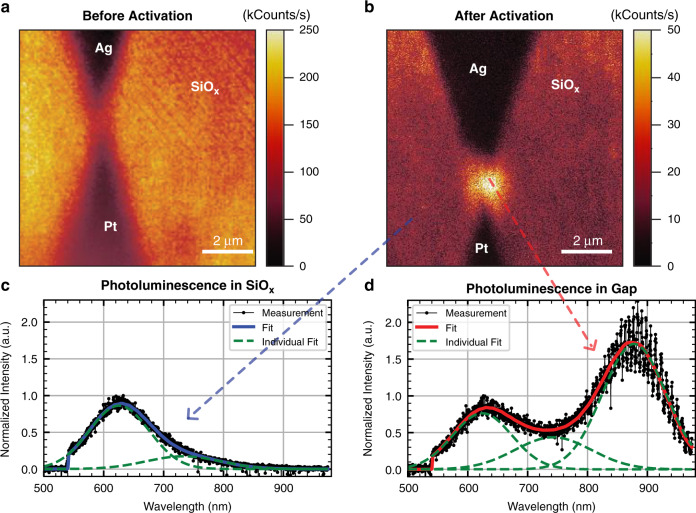
PL measurement indicating the creation of luminescent defects created by resistive switching. **a** Confocal PL scan of the pristine device. The laser power is 1 µW with an integration time of 1 ms per pixel. **b** Confocal PL scan of the device after activation and switching. The laser power is 300 nW with an integration time of 0.5 ms per pixel, which yields an approximately 6 times weaker PL for the SiO_x_ (25 kHz in **b** and 160 kHz in **a**). **c** PL spectrum taken at a SiO_x_ spot outside of the gap. Gaussian fits are used to decompose the spectrum. Two spectral components (green dashed lines) are identified that contribute to the overall emission. **d** PL spectrum taken in the gap. A new dominant emission around 875 nm is found. This is best seen from the fit with Gaussian functions. Note that the intrinsic peak at 625 nm in both c and d are normalized to the same amplitude

To locate the origin of the luminescence and to show that it stems from the gap, PL scans are performed on an APS before and after electrically activating the device. The APS is optically excited with a 515 nm diode laser. The details of the optical setup are given in Supplementary Section II. First, a PL confocal scan is carried out on a pristine device that has never been switched before. The image displayed in Fig. 5a features two dark triangular regions that are the metal contacts to the antenna structure. The relatively bright photoluminescence background observed when the laser excites the matrix originates from native luminescent defects in the as-sputtered a-SiO_x_ cladding layer, which is naturally oxygen-rich (see the XPS measurement in Supplementary Section V). After a few cycles of resistive switching, the PL confocal scan is repeated, and the result is shown in Fig. 5b. Compared to the as-sputtered SiO_x_, a much brighter PL emission center appears between the metal contacts, indicating that new luminescent sites are created in the gap after resistive switching. Please note that a lower laser power is applied for PL excitation in Fig. 5b to avoid excessive photobleaching. Also note that the PL spectrum does not perfectly match the EL spectrum since the contributing population and nature of the defects may differ between electron injection and photo-excitation. Similar EL and PL differences have been reported in antenna-enhanced light-emitting devcies^23^ and are discussed in more detail in the Supplementary Section IX.

To confirm the presence of new species formed upon repetitive switching, PL spectra of the as-sputtered SiO_x_ outside of the gap and in the gap after resistive switching are compared in Fig. 5c and d, respectively. The spectra of the as-sputtered SiO_x_ cladding layer before activation measured outside of the gap and in the gap are similar to each other and resemble the one depicted in Fig. 5c, see Supplementary Section VI. After resistive switching, an additional dominant PL peak around 875 nm is revealed in the spectrum taken in the gap. This contribution is new and has not been observed in the gap of any pristine devices. It is also distinct from the ones observed in the as-sputtered SiO_x_ cladding and thus must originate from additional luminescent sites induced by resistive switching. To decompose the dominant contributions to the respective spectra, the curves are fitted by Gaussian functions. Besides the new peak at 875 nm, the decomposition reveals two peaks centered at ~625 and 745 nm in both spectra.

Furthermore, by comparing the two spectra, it is evident that the amplitude of the peak at 745 nm revealed by the decomposition is increased by a factor of 3 after switching compared to the spectrum of the SiO_x_ cladding. This suggests that the PL emitted by the activated gap results from the additive contribution of new luminescence centers created in the oxide during the resistive switching process (875 nm peak) and additional pre-existing species emitting at 745 nm. Importantly, these PL peaks overlap the broad EL spectrum observed in Fig. 3d, indicating that the luminescence of the EL and PL originates from the same luminescent structures.

### Investigation under reverse-bias switching

To determine what type of luminescent sites cause the light emission in the APS, additional EL and PL measurements with a reverse-bias are performed on a pristine device (see Supplementary Section VII). Unlike forward-bias switching, Ag oxidation and Ag filament formation do not occur in reverse-bias switching^24^. Hence the matrix remains free of Ag under this biasing condition. By conducting a reverse-bias switching measurement, the luminescence from metal species such as silver nanoclusters^25^ can be excluded.

Reverse biasing required much higher voltages and showed weaker photon emission (see Supplementary Section VII). Nevertheless, very similar EL and PL spectra compared to forward-bias switching are observed. Notably, a similar switching–induced PL spectral change is observed under both reverse- and forward biasing, which indicates the creation of luminescent sites in either operation mode by the same processes. We conclude that the underlying origin of photons is the same for both forward and reverse bias. Correspondingly, as no silver is present in the gap during reverse switching, we argue that the light emission does not originate from silver nanoclusters^25^ but from defects in the SiO_x_ matrix.

## Discussion

Following the previous two sections, it is now understood that the luminescence stems from a structural change of the SiO_x_ matrix within the gap. The role of the silver in the APS is to lower the switching voltage and to make the luminescence more controllable and intense. Here we discuss what luminescent sites are the most likely origin of the observed light emission and propose a photon emission mechanism.

In the case of reverse-biasing, the APS switches by a controlled dielectric breakdown in the SiO_x_ matrix. Such mechanisms are known from OxRAM-type memristors^26^, where operation is attributed to the creation and aggregation of oxygen vacancies^27–30^. Whereas high voltages are required in the reverse-bias case, the creation and aggregation of oxygen vacancies into oxygen vacancy clusters also occur in the case of forward-bias experiments at lower voltage in the presence of the Ag filament. It has been reported that the introduction of metal species (Ag in our device) lowers the energy barrier to create oxygen vacancies^31^, making the resistive switching more controllable with a reduced switching voltage^32^.

The formation and aggregation of the oxygen vacancy clusters in forward-switching can be understood as follows: Intrinsic defects in deposited amorphous SiO_x_ (such as wide O–Si–O bond angles) or strain introduced defects during filament growth and dissolution could act as precursors^33,34^. These precursors lower the energy barrier for the creation of oxygen vacancies. Once created, the barrier to create additional oxygen vacancies closeby is further reduced, which leads to an aggregation of oxygen vacancies^29^ into oxygen vacancy clusters. Oxygen vacancies are known to be luminescent upon electrical or optical excitations^35^. However, the reported emission spectra typically feature signatures located in the UV to visible spectral regions and do not match our observations. Nevertheless, the creation and aggregation of oxygen vacancies into oxygen vacancy clusters generate locally silicon-rich regions within the SiO_x_ matrix. Luminescence spectra of silicon-rich oxide matching our observed EL and PL peak wavelengths have been reported in various references^36–39^. Thus, we attribute the photon emission of the APS to defects in locally silicon-rich regions^39,40^ formed by the creation and aggregation of oxygen vacancies into oxygen vacancy clusters upon electrical activation of the device.

Photon emission in these Si-rich clusters matching the EL and PL wavelength of the APS shown in Fig. 3d and Fig. 5d can be further divided into radiative recombination in Si nanocores and Si–O compound clusters^37,41,42^. The latter appear either as clusters formed at the interface layer of Si nanocores^37,38,41,43^ or as distinct clusters in silicon-rich regions^37,44,45^. The photon emission in the APS is more likely attributed to Si–O compound clusters for two reasons. First, the reported PL of Si nanoclusters matching our PL wavelength is generally attributed to the Si–O interface layer, whereas PL from the Si nanocore is only reported in the blue^37,41,46,47^. Second, we conducted PL lifetime measurements (plots are given in Supplementary Section VIII), revealing that the switching-induced PL peak at ~875 nm features a lifetime of around 8 ns. As this measured lifetime is around ten times longer than the reported lifetimes from Si nanocore transitions^41,42^, they can likely be excluded as the origin of the APS light emission. However, the lifetime of Si–O compound interfaces is reported to be 10^4^ to 10^5^ times longer than our measured decay ^37,41,47^. Such a discrepancy can be reasonably explained by the drastic shortening of the lifetime due to the Purcell effect, which is investigated by our LDOS simulation shown in Supplementary Section IV. Once created, this type of Si-rich defects can then be excited by tunneling electrons transported in the gap and can be radiatively recombined^48^ and thus represent the origin of light in the memristive atomic photon source.

The photon emission by memristive switching is more generic and not limited to the Ag/SiO_x_/Pt material system. We have already observed a much stronger photon emission from memristors with Ag/PMMA/Ag layer stacks. We believe the emission is also from the memristive switching-induced luminescent centers and atomic rearrangements of the Ag filament. However, the luminescence mechanism in this material system needs further investigation to confirm our hypothesis. In any case, the new memristive light emitters have the potential to enable many new applications e.g. related to neurons, which upon activation, fire and emit.

## Conclusion

In conclusion, photon emission is reported during the resistive switching process of Ag/amorphous -SiO_x_/Pt atomic scale memristors. Our investigations suggest that the emission stems from electroluminescent Si-rich defects generated during resistive switching and atomic rearrangements of the conductive filament. The findings are supported by electroluminescence and confocal photoluminescence measurements. The optical response of the atomic switch can be optimized for a high radiation efficiency by introducing an asymmetrical Ag-Pt antenna as well as by maximizing the LDOS enhancement with the help of Ag filament formation. The engineered photon source discussed here features an atomic-sized footprint and a straightforward and scalable fabrication process. As the emitted photons are associated with a resistive state change, our findings can be exploited in optical memristive neural networks to identify weight changes from the corresponding memristor.

## Materials and methods

The details of sample fabrication, experimental setup and numerical simulations are provided in Supplementary Information section I, II, and IV, respectively.

## Supplementary information

Supplementary Information_Atomic Scale Memristive Photon Source

## References

[1] Zhou ZP, Yin B, Michel J (2015). On-chip light sources for silicon photonics. Light Sci. Appl..

[2] Sun C (2015). Single-chip microprocessor that communicates directly using light. Nature.

[3] Kozma P (2014). Integrated planar optical waveguide interferometer biosensors: a comparative review. Biosens. Bioelectron..

[4] Emboras A (2016). Atomic scale plasmonic switch. Nano Lett..

[5] Shen HB (2019). Visible quantum dot light-emitting diodes with simultaneous high brightness and efficiency. Nat. Photonics.

[6] Shirasaki Y (2013). Emergence of colloidal quantum-dot light-emitting technologies. Nat. Photonics.

[7] Senellart P, Solomon G, White A (2017). High-performance semiconductor quantum-dot single-photon sources. Nat. Nanotechnol..

[8] Parzefall M (2015). Antenna-coupled photon emission from hexagonal boron nitride tunnel junctions. Nat. Nanotechnol..

[9] Kern J (2015). Electrically driven optical antennas. Nat. Photonics.

[10] Qian HL (2018). Efficient light generation from enhanced inelastic electron tunnelling. Nat. Photonics.

[11] Uskov AV (2016). Excitation of plasmonic nanoantennas by nonresonant and resonant electron tunnelling. Nanoscale.

[12] Strukov DB (2008). The missing memristor found. Nature.

[13] Waser R, Aono M (2007). Nanoionics-based resistive switching memories. Nat. Mater..

[14] Menzel S (2013). Switching kinetics of electrochemical metallization memory cells. Phys. Chem. Chem. Phys..

[15] Hasegawa T (2012). Atom/ion movement controlled devices for beyond Von‐Neumann computers. Adv. Mater..

[16] Cheng BJ (2019). Ultra compact electrochemical metallization cells offering reproducible atomic scale memristive switching. Commun. Phys..

[17] Kim KH (2012). A functional hybrid memristor crossbar-array/CMOS system for data storage and neuromorphic applications. Nano Lett..

[18] Wang ZR (2020). Resistive switching materials for information processing. Nat. Rev. Mater..

[19] Emboras A (2018). Atomic scale photodetection enabled by a memristive junction. ACS Nano.

[20] Zhu YB (2021). Light-emitting memristors for optoelectronic artificial efferent nerve. Nano Lett..

[21] Lanza M (2019). Recommended methods to study resistive switching devices. Adv. Electron. Mater..

[22] Cheng BJ (2021). Threshold switching enabled sub-pW-leakage, hysteresis-free circuits. IEEE Trans. Electron Devices.

[23] Cui LJ (2021). Thousand-fold increase in plasmonic light emission via combined electronic and optical excitations. Nano Lett..

[24] Tsuruoka T (2010). Forming and switching mechanisms of a cation-migration-based oxide resistive memory. Nanotechnology.

[25] Lee TH, Gonzalez JI, Dickson RM (2002). Strongly enhanced field-dependent single-molecule electroluminescence. Proc. Natl Acad. Sci. USA.

[26] Menzel, S. & Waser, R. Mechanism of memristive switching in OxRAM. In: *Advances in Non-Volatile Memory and Storage Technology* 2nd edn (eds Magyari-Köpe, B. & Nishi, Y.) (Amsterdam: Elsevier, 2019).

[27] Mehonic A (2016). Nanoscale transformations in metastable, amorphous, silicon‐rich silica. Adv. Mater..

[28] Nadimi E (2010). Single and multiple oxygen vacancies in ultrathin SiO_2_ gate dielectric and their influence on the leakage current: an ab Initio investigation. IEEE Electron Device Lett..

[29] Padovani A (2017). A microscopic mechanism of dielectric breakdown in SiO_2_ films: an insight from multi-scale modeling. J. Appl. Phys..

[30] Wang YF (2013). Resistive switching mechanism in silicon highly rich SiO_x_ (x< 0.75) films based on silicon dangling bonds percolation model. Appl. Phys. Lett..

[31] O’Hara A, Bersuker G, Demkov AA (2014). Assessing hafnium on hafnia as an oxygen getter. J. Appl. Phys..

[32] Huang X (2018). Configurable ultra-low operating voltage resistive switching between bipolar and threshold behaviors for Ag/TaO_x_/Pt structures. Appl. Phys. Lett..

[33] Gao DZ, El-Sayed AM, Shluger AL (2016). A mechanism for Frenkel defect creation in amorphous SiO_2_ facilitated by electron injection. Nanotechnology.

[34] Ichihara R (2019). Investigation of switching-induced local defects in oxide-based CBRAM using expanded analytical model of TDDB. IEEE Trans. Electron Devices.

[35] Salh, R. Defect related luminescence in silicon dioxide network: a review. In *Crystalline Silicon-Properties and Uses* (ed Basu, S.) 135–172 (Rijeka: IntechOpen, 2011).

[36] Meldrum A (2006). Photoluminescence in the silicon-oxygen system. J. Vac. Sci. Technol. A Vac. Surf. Films.

[37] Rodríguez JA (2014). Emission mechanisms of Si nanocrystals and defects in SiO_2_ materials. J. Nanomaterials.

[38] Ni ZY (2019). Silicon nanocrystals: unfading silicon materials for optoelectronics. Mater. Sci. Eng. R Rep..

[39] He CL (2013). Tunable electroluminescence in planar graphene/SiO_2_ memristors. Adv. Mater..

[40] Anutgan T (2017). Electroformed silicon nitride based light emitting memory device. Appl. Phys. Lett..

[41] Kanemitsu Y (1994). Luminescence properties of nanometer-sized Si crystallites: core and surface states. Phys. Rev. B.

[42] Roy S (2010). Fluorescence lifetime analysis and fluorescence correlation spectroscopy elucidate the internal architecture of fluorescent silica nanoparticles. Langmuir.

[43] Yao J (2012). In situ imaging of the conducting filament in a silicon oxide resistive switch. Sci. Rep..

[44] Aceves-Mijares M (2012). On the origin of light emission in silicon rich oxide obtained by low-pressure chemical vapor deposition. J. Nanomaterials.

[45] López-Estopier, R., Aceves-Mijares, M. & Falcony, C. Cathodo-and photo-luminescence of silicon rich oxide films obtained by LPCVD. In *Cathodoluminescence* (ed Yamamoto, N.) 253–272 (Rijeka: IntechOpen, 2012).

[46] Wilcoxon JP, Samara GA, Provencio PN (1999). Optical and electronic properties of Si nanoclusters synthesized in inverse micelles. Phys. Rev. B.

[47] Dohnalová K (2010). White-emitting oxidized silicon nanocrystals: discontinuity in spectral development with reducing size. J. Appl. Phys..

[48] Lee TH, Dickson RM (2003). Discrete two-terminal single nanocluster quantum optoelectronic logic operations at room temperature. Proc. Natl Acad. Sci. USA.

