# Supplementary material for: Atomic scale memristive photon source
Source: Light Sci Appl. 2022 Mar 29;11:78. doi: 10.1038/s41377-022-00766-z (PMC8964763; doi:10.1038/s41377-022-00766-z)
Supplement: Supplementary file 1 — Supplementary Information_Atomic Scale Memristive Photon Source [file 41377_2022_766_MOESM1_ESM.pdf]

Supplementary Information for

Atomic Scale Memristive Photon Source

Bojun Cheng[1✉†], Till Zellweger[1†], Konstantin Malchow[2†], Xinzhi Zhang[1],

Mila Lewerenz[1], Elias Passerini[1], Jan Aeschlimann[3], Ueli Koch[1], Mathieu Luisier[3],

Alexandros Emboras[3], Alexandre Bouhelier[2] and Juerg Leuthold[1✉]

[1]ETH Zurich, Institute of Electromagnetic Fields, Zurich 8092, Switzerland

[2]Laboratoire Interdisciplinaire Carnot de Bourgogne, UMR 6303 CNRS, Université de Bourgogne Franche-Comté, Dijon 21078, France

[3]ETH Zurich, Integrated Systems Laboratory, Zurich 8092, Switzerland

✉Corresponding author: bojun.cheng@ief.ee.ethz.ch; leuthold@ethz.ch

[†]These authors contributed equally: Bojun Cheng, Till Zellweger, Konstantin Malchow

I. Sample fabrication

To achieve a high collection efficiency by the high NA objective, we use calibrated 170 µm thick glass coverslips as a sample substrate. A layer of 30 nm Ag is deposited on top of the entire surface. Another 15 nm Au is subsequently deposited on top to protect the Ag from oxidation. We use e-beam lithography and Ar ion milling to fabricate the Ag antenna and remove the excess Ag. We perform a second step of e-beam lithography to pattern the Pt antenna. 50 nm Pt is deposited by e-beam evaporation, and the subsequent structure is finalized by a liftoff process. Finally, the 60 nm amorphous SiO_x cladding layer is deposited by RF sputtering and serves as the switching layer at room temperature. We use a SiO_2 target in an Ar environment with a pressure of 2 µbar. The RF power is 600 W and the deposition rate is ~ 0.46 nm·s^{-1}. The SiO_x cladding layer is oxygen-rich, as shown by the XPS measurements in Supplementary Section V. The samples presented in this work were fabricated at the cleanroom facilities of the Binnig and Rohrer Nanotechnology Center (BRNC).

II. Measurement setup

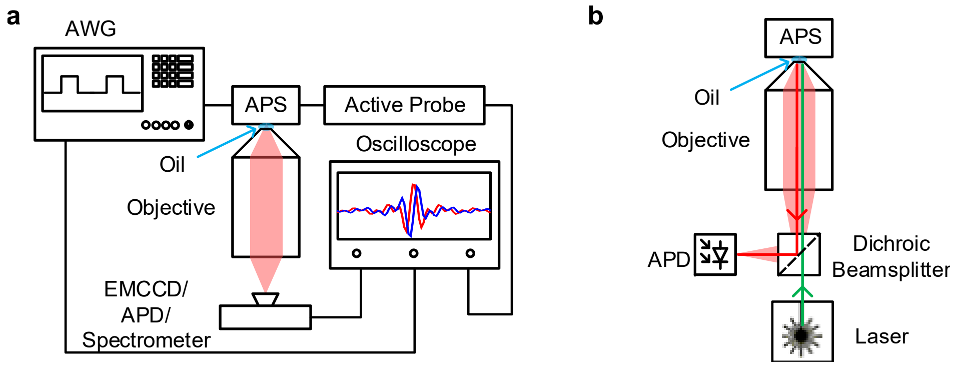

Fig. S1 | Measurement setup. a, Setup for the electro-optical measurements. In the illustration, an electron-multiplying CCD (EMCCD) camera is used as a detector. It can be exchanged by an APD or spectrometer by selecting the desired output port of an inverted microscope. **b**, Setup for PL measurements. The APS is excited by a strongly focused 515 nm laser that can be operated in CW mode or in picosecond pulsed mode with a selectable repetition rate (20 MHz to 80 MHz). A dichroic beam splitter filters the excitation wavelength and sends the PL response to an avalanche photodiode counting module (APD). The latter can be connected to a Time-Correlated Single Photon Counting module (TSCPC) to determine the PL lifetime.

The setup used for the electric and optical characterizations is shown in Fig. S1a. The sample containing the device under test (APS) is placed on top of an inverted microscope (Eclipse, Nikon) and is imaged using an oil immersion objective (100x, 1.49 NA, Nikon). The emitted light is then sent either to an electron-multiplying EMCCD camera (Newton, Andor Technology), an APD (SPCM-AQRH, Excelitas), or a spectrometer (Shamrock 300i, Andor) by choosing the desired output port. A piezoelectric translation stage (PI) allows precise sample positioning and is commanded by a control electronic (R9, RHK). The APS is connected to a high-speed active probe (Model 35, GGB Industries) with 1.25 MΩ internal resistance. A voltage signal from an arbitrary waveform generator (AWG) is fed through a probe to the APS. The active probe with high internal resistance also limits the current from getting too high and electrically damaging the APS. The high-speed probe is connected to an oscilloscope to measure the current from the APS.

The setup used for the photoluminescence is shown in Fig. S1b. The inverted microscope allows us to perform photoluminescence measurements by inserting a dichroic beam splitter (Chroma) in the microscope and using a diode laser at 515 nm wavelength excitation. The units to perform the lifetime measurements are not represented in the figure.

III. Optical wide-field images

The image shown in Fig. 1c of the main text is the result of two images numerically overlayed. First, an optical image taken in transmission with external illumination and with no bias applied is recorded, as shown in Fig. S2a. Then the external illumination is switched off, and a voltage bias is applied. The corresponding EL wide-field optical image is shown in Fig. S2b. The bright spot appears at the gap between Ag and Pt. The *I* - *V* sweep curve measured while obtaining the EL image of Fig. S2b is plotted in Fig. S2c.

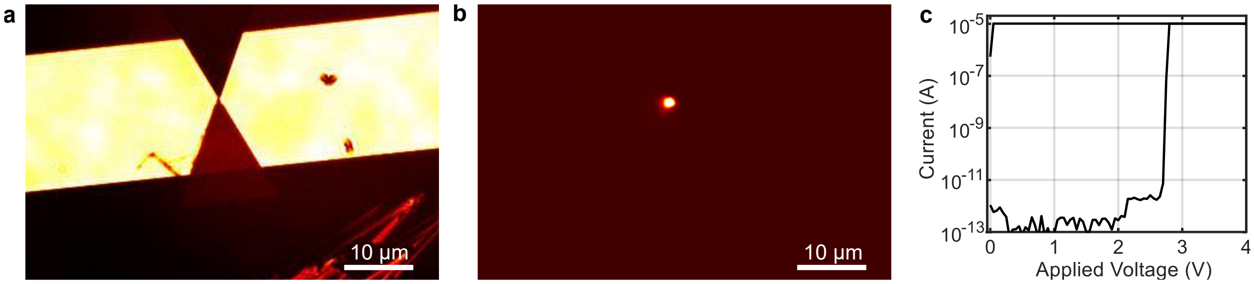

Fig. S2 | Optical images of the APS and the accompanying *I* - *V* sweep. a, Transmission wide-field micrograph of the APS with external diascopic illumination and no bias voltage applied. The glass substrate and SiO_x cladding are transparent and thus appear brighter. The electrodes are non-transparent and appear darker. The Ag electrode is at the top and the counter Pt electrode at the bottom. The antenna located at the extremities cannot be observed at this magnification. **b**, Wide-field image of the APS under operation. Integration time is set to 30 s. The source of light is located in the gap between Ag and Pt. **c**, The *I* - *V* sweep curve measured while obtaining the EL image of b. Upon switching to the low resistance state, a compliance current of I_{cc} = 10 µA limits the maximum current flowing through the device.

IV. Nanoantenna simulation

Simulation design. To investigate and understand the role of the nanoantennas and the metallic filament on the photon emission of the APS, we conduct 3D finite element method (FEM) simulations with the COMSOL Multiphysics software. The quantum efficiency (*QE*) of the system is calculated using parameters extracted from the FEM simulation. We show the simulated architecture in Fig. S3. The dimensions and spacing of the silver and platinum nanoantennas are obtained from the SEM image shown in Fig. 1b of the main section. We model the Ag filament as a cylindrical Ag rod with radius and tip radius *r* and a filament-antenna gap d. A x-polarized electric dipole source (red arrow) is placed at the center of the gap.

Using this simulation model, two important measures, P_{tot} (the total radiated power from the electric dipole) and P_{z-} (the far-field power radiated towards the objective located in the negative z-plane), are extracted. Using these values, we can express the LDOS enhancement L as well as the fraction of the power radiated to the z-half-space, referred to as the radiation efficiency η_{z-}:

$$\eta_{z-} = \frac{P_{z-}}{P_{\mathrm{tot}}} \tag{1}$$

$$L = \frac{P_{\mathrm{tot}}}{P_0} \tag{2}$$

where P_0 is the radiated power from the same electric dipole in vacuum.

To express the total quantum efficiency of the emissive defects, the characteristics of a quantum emitter have to be taken into account. In the two-level approximation, such an emitter in vacuum features a radiative and intrinsic non-radiative decay rate defined as γ_{r}^{0} and as $\gamma_{\mathrm{nr}}{}^{0}$, resulting in an internal quantum efficiency of $QE_0 = \gamma_{\mathrm{r}}^{0}/(\gamma_{\mathrm{nr}}{}^{0} + \gamma_{\mathrm{r}}^{0})$ [1]. In the presence of a nanoantenna, the local optical mode density (LDOS) is altered, which leads to an enhanced total decay rate γ_{tot}. γ_{tot} is linked to the radiative

decay rate in vacuum γ_r^0 by the LDOS enhancement[1, 2] $L = \gamma_{tot}/\gamma_r^0$. The total decay rate of the dipole consists of non-radiative and radiative contributions $\gamma_{tot} = \gamma_r + \gamma_{nr}$, where γ_r is the radiative decay rate observable in the far field and γ_{nr} is an additional non-radiative decay rate caused by coupling to non-radiative modes of the optical antenna[3]. In summary, γ_{tot} can be expressed as

$$\gamma_{tot} = \gamma_r + \gamma_{nr} = \gamma_r^0 \cdot L \tag{3}$$

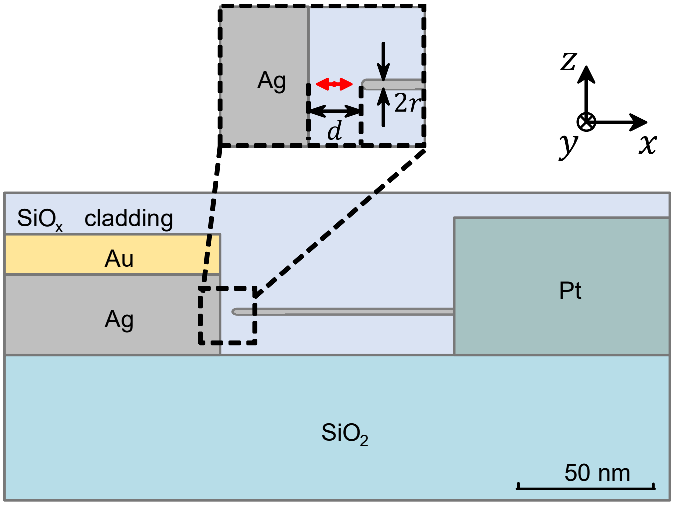

Fig. S3 | Schematic of the model structure for 3D FEM simulation. Ag and Pt antennas are buried inside sputtered SiO_x. The thicknesses and dimensions of Au, Ag, Pt, and SiO_x are the same as used in fabrication. The Ag filament grown during the resistive switching is modeled by a cylindrical rod with radius and tip radius *r*, leaving a filament-antenna gap *d* between the filament and the Ag antenna. An x-polarized electric dipole is placed at the center of the gap (illustrated by the red arrow). Radiation efficiency and LDOS enhancement are calculated numerically in this model. The complex refractive indices of Au, Ag and Pt are extracted from separate ellipsometry measurements. The SiO_2 substrate and SiO_x cladding are modeled with a constant refractive index of 1.4.

The quantum efficiency of the emitter coupled to the antenna can be expressed as:

$$QE = \frac{\gamma_r}{\gamma_r + \gamma_{nr} + \gamma_{nr}{}^0} \tag{4}$$

As aforementioned, we can only observe the fraction of photons emitted towards the z- half-space, given by the radiation efficiency η_{z-}:

$$\eta_{z-} = \frac{\gamma_{r,z-}}{\gamma_{tot}} = \frac{\gamma_{r,z-}}{\gamma_r + \gamma_{nr}} \tag{5}$$

Where $\gamma_{r,z-}$ is the fraction of γ_r observed in the z-half-space. To account for this parameter, a separate quantum efficiency QE_{z-} can be defined:

$$QE_{z-} = \frac{\gamma_{r,z^-}}{\gamma_r + \gamma_{nr} + \gamma_{nr}{}^0} = \frac{\gamma_{tot} \cdot \eta_{z^-}}{\gamma_{tot} + \gamma_{nr}{}^0} \tag{6}$$

Under the assumption that the quantum emitter acts as a point-dipole with a time-varying dipole moment, L and η_{z-} are equal to their counterpart in the classical electromagnetism picture given in equations 1 and 2[1, 3]. Both γ_{tot} and η_z-can thus directly be extracted from the simulations and be used to estimate their effect on QE_{z-}.

$$QE_{z-} = \frac{\gamma_{tot} \cdot \eta_{z^-}}{\gamma_{tot} + \gamma_{nr}^0} = \frac{\gamma_r^0 L}{\gamma_r^0 L + \gamma_{nr}^0} \cdot \eta_{z^-} \tag{7}$$

According to the literature of Si-O compound cluster interfaces, those luminescent sites typically have a long radiative lifetime in the order of hundreds of microseconds[4], which causes a very low quantum efficiency QE_0 due to the Auger effect[5]. It can thus be assumed that $\gamma_{\mathrm{nr}}^0 \gg \gamma_{\mathrm{r}}^0$. In the range of the simulated LDOS enhancement we can further assume that $\gamma_{\mathrm{nr}}^0 \gg \gamma_{\mathrm{r}}^0 L$. We can thus simplify the expression of the quantum efficiency as follows:

$$QE_{z-} = \frac{\gamma_{\mathrm{r}}^0 L}{\gamma_{\mathrm{r}}^0 L + \gamma_{\mathrm{nr}}^0} \cdot \eta_{z^-} \approx \frac{\gamma_{\mathrm{r}}^0}{\gamma_{\mathrm{nr}}^0} L \cdot \eta_{z^-} \tag{8}$$

Accordingly, the quantum efficiency $QE_{z-}$of a Si-O compound cluster coupled to an antenna can be optimized by increasing the factor $L \cdot \eta_{z^-}$.

As the dimensions of the filament cannot be determined accurately with SEM or other techniques, the influence of the filament-antenna gap (d) or the filament radii (r) on η_{z-} and L is simulated and investigated in the following for a range of d and r.

Influence of the filament-antenna gap. As discussed in the main section of this paper, the simulations showed that the presence of an Ag filament only causes a small decrease in the factor $L \cdot \eta_{z^-}$ (see Fig. 4 in the main text). Here the influence of the filament as well as the filament-antenna gap d on this factor is described in more detail. In Fig. S4, the simulated radiation efficiency η_{z-} as well as the LDOS enhancement are calculated for various gap sizes $d = 3{,}5{,}7\ \mathrm{nm}$ with a fixed filament radius $r = 0.5\ \mathrm{nm}$ and compared to the same simulation without filament.

Fig. S4a reveals a decrease of η_{z-}, with the introduction of the filament. The decrease is more pronounced for smaller filament-antenna distances. This can be explained by a quenching effect due to the enhanced excitation of non-radiative higher-order modes of the filament, which is promoted by smaller separations between the emitter and the filament[3, 6, 7]. This leads to a greatly enhanced γ_{nr} in equation 5. On the other hand, as shown in Fig. S4b, the metallic filament also causes an overall very high LDOS enhancement, thereby largely compensating for the decreasing η_{z-}. We can thus conclude that the optimized optical antenna with or without filament allows obtaining a higher quantum efficiency at the EL wavelength of the APS.

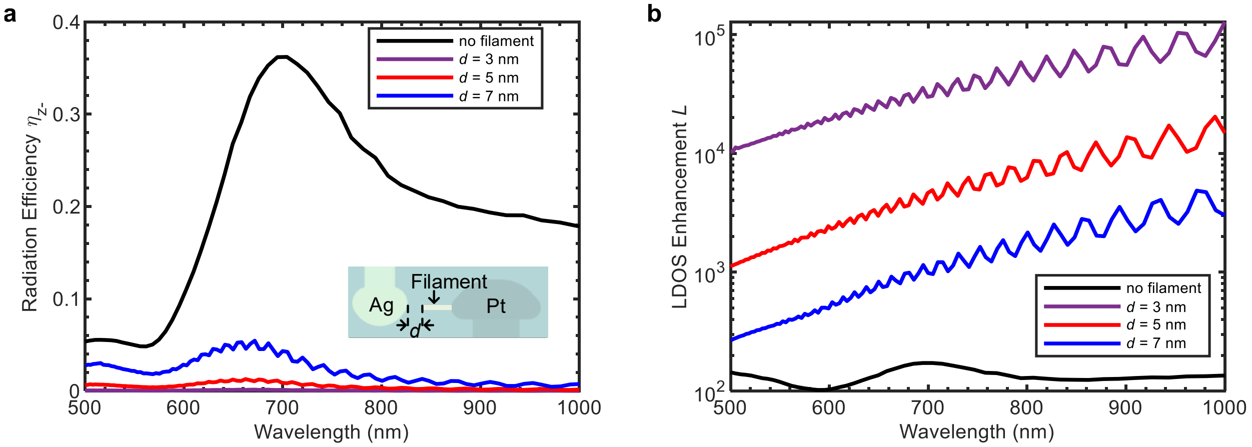

Fig. S4 | Simulation for fixed filament radius and varying gap sizes. a, Simulated radiation efficiency (η_{z-}) in *z*-direction with fixed filament radius *r* = 0.5 nm. Smaller filament-antenna gap shows lower η_{z-}. Inset, the structure of the Ag and Pt nanoantenna used in the simulation. **b**, Simulated LDOS enhancement (*L*). Smaller filament-antenna gap shows higher *L*. The influence of filament-antenna gap on η_{z-} and *L* are opposite and limits the influence of the filament in its product, see Fig. 4b in the article.

Influence of the filament shape. In Fig. S5, the factor L, η_{z^-}, and their product are depicted for various filament radii of $r = 0.3, 0.5, 1\ \mathrm{nm}$ with a fixed filament-antenna gap $d = 5\ \mathrm{nm}$. The simulation shows a decrease of η_{z^-} (Fig. S5a), an increase of L (Fig. S5b), and a slight decrease of $L \cdot \eta_{z^-}$ (Fig. S5c) with

increasing filament radius. Fluctuations of L and η_{z^-} as a function of wavelength are also observed and are more pronounced with increasing filament radius.

To elucidate the origin of the fluctuations and the decrease of $L \cdot \eta_{z^-}$, the amplitude of the electric field E_{norm} around the gap of the APS is simulated for a filament-antenna gap $d = 7$ nm and a filament radius $r = 0.5$ nm at wavelength $\lambda = 700$ nm, shown in Fig. S5d. A standing wave is clearly visible along the filament, which is caused by the excitation of higher-order modes and their reflection at the interfaces (formation of a standing wave). As discussed above, the excitation of these higher-order modes causes luminescence quenching by means of decreasing the radiation efficiency. We can thus conclude that an increase of the filament radius enhances the excitation of these modes and leads to a decrease of the factor $L \cdot \eta_{z^-}$. Similarly, the observed fluctuations at larger radii are likely caused by the pronounced standing waves inside the filament.

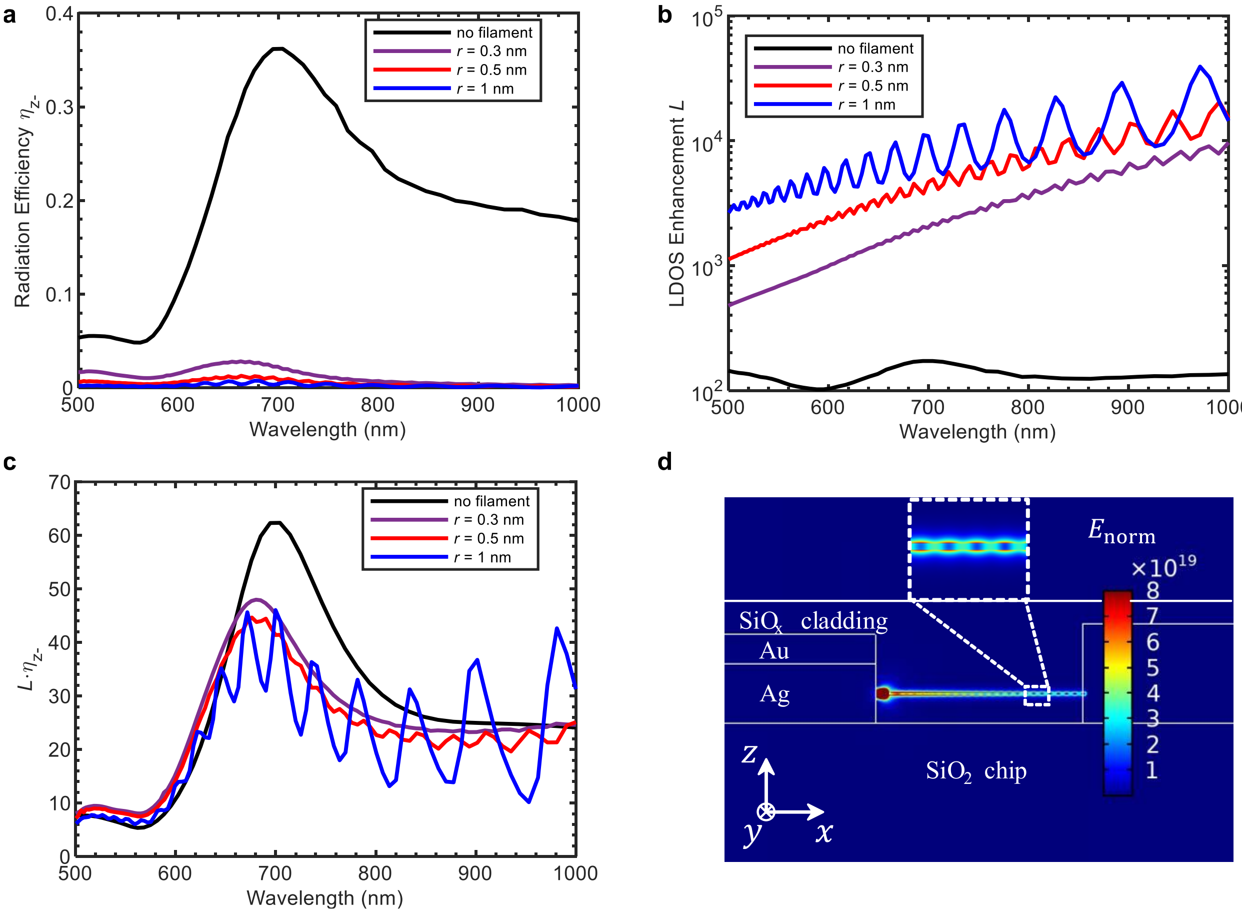

Fig. S5 | Simulation for fixed gap sizes radius and varying filament. a, Simulated radiation efficiency (η_{z-}) in *z*- direction with fixed filament-antenna gap *d* = 5 nm as a function of the filament radius *r*. The thicker filament features lower η_{z-}. **b**, Simulated LDOS enhancement (*L*) as a function of the filament radius *r*. The thicker filament features higher *L*. **c**, Simulated *L* η_{z-}a function of filament radius. The thicker filament features slightly lower *L* η_{z-}. **d**, Simulated electric field distribution for a filament with *d* = 7 nm and *r* = 0.5 nm at wavelength λ = 700 nm. The region with maximum magnitude is the filament-antenna gap with a dipole source in the center. A strong standing wave is observed inside the silver filament, which explains the oscillatory behavior of the spectrum.

V. XPS analysis of SiO_x

The X-ray photoelectron spectroscopy (XPS) gives the ratio of Si and O atoms of the sputter-deposited SiO_x cladding and the glass substrate. The recorded XPS spectra on the two interfaces are presented in Fig. S6**.** A precise ratio between the atomic constituents is given in Table S1. From the Table, one can derive that both the glass substrate and the deposited SiO_x cladding have an off-stochiometric oxygen ratio above 1:2 at the surface. This is due to the oxygen in the atmosphere. Once the surface layer is locally etched by a 500 keV X-ray, the ratios change. After 5 mins etch, the Si:O ratio becomes close to 1:2 in the glass substrate. In the case of the sputtered SiO_x, a Si:O ratio of 29.1:70.9 is measured, indicating that the layer is oxygen-rich.

Table S1 | Si to O ratio extracted from the XPS Measurement.

	Deposited SiO_x Cladding	Glass Substrate
Surface	26:74	31:69
After 1min 500 keV etch	28.3:71.7	31.8:68.2
After 5min 500 keV etch	29.1:70.9	33.6:66.4

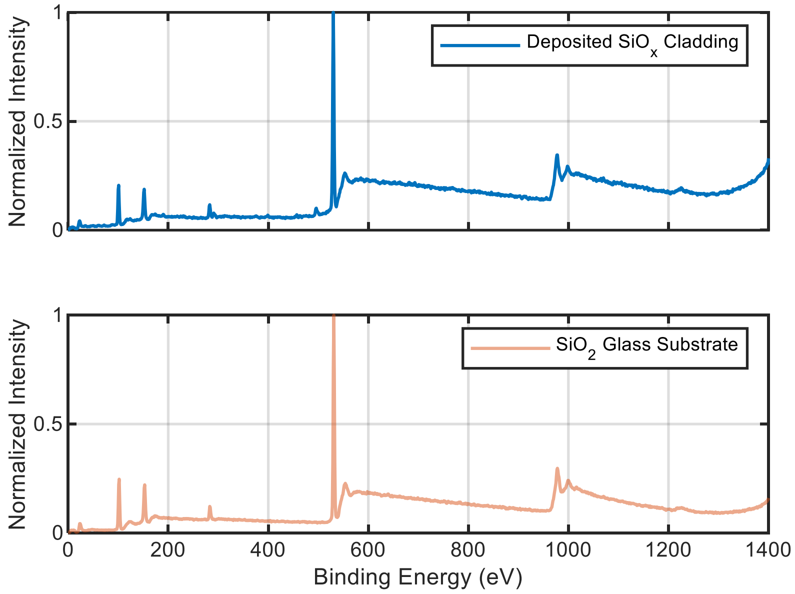

Fig. S6 | XPS Measurement of the SiO_x. a, XPS Measurement of the sputtered SiO_x cladding. **b**, XPS Measurement of the glass substrate without SiO_x sputtering.

VI. PL of the pristine device

In this section, additional PL measurements of a device before switching are presented. The PL spectra of the gap between the Ag and Pt electrode as well as from the SiO_x are measured and compared to exclude any luminescent sites possibly present in the gap before the initial switching.

The PL scan of the pristine device is shown again in Fig. S7a for reference. The PL spectra are measured both in the gap and the SiO_x cladding outside of the gap. The raw measurements are shown in Fig. S7b. The PL intensity in the gap is lower because of the local absorption imparted by the metal electrodes. For a better comparison, the normalized PL spectra are given in Fig. S7c. The PL spectra measured from the deposited SiO_x cladding and at the gap are almost identical before activation, indicating that similar native defects are present in the switching matrix. As discussed in the main text, the PL spectrum of the SiO_x

cladding is similar before and after activation, while the PL spectra from the gap differ significantly after activation.

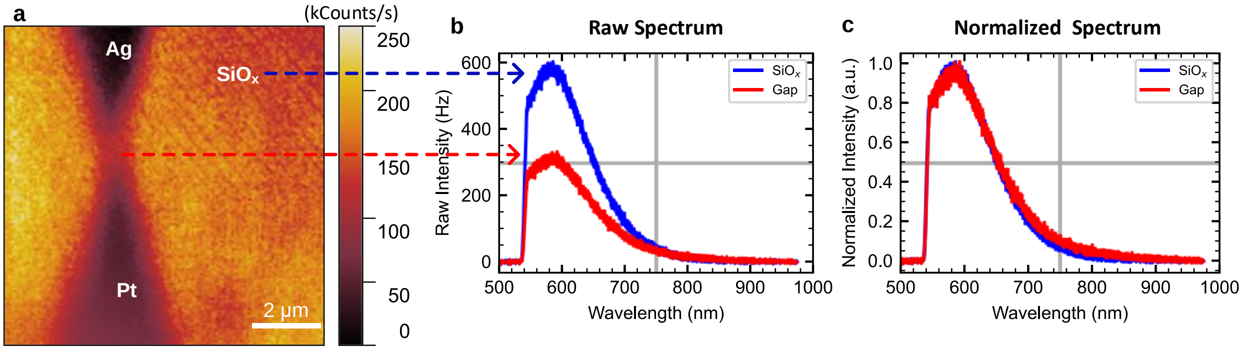

Fig. S7 | Photoluminescent measurement of a pristine device. **a**, Confocal PL scan of the pristine device. **b**, Raw data of PL spectra from deposited SiO_x and from within the gap. **c**, Normalized PL spectrum of the gap and the SiO_x cladding. The plots show that the PL spectra are almost identical at the gap and the SiO_x cladding outside of the gap before activation.

VII. Reverse-bias switching

A reverse-bias resistive switching experiment is performed on a pristine device. Under this biasing polarity, the growth of a silver filament is inhibited. The experiment shows similar albeit less intense EL and PL spectral responses as observed under a forward-bias resistive switching.

The measurement is performed by applying a negative bias to the Ag electrode of a pristine APS device. The negative voltage prevents Ag oxidation and Ag filament formation[8]. Consequently, a much higher voltage is required to induce resistive switching (dielectric breakdown). The EL spectrum measured during reverse-bias switching is plotted in red in **Fig. S8**a. The EL spectrum of a forward-bias switching is plotted in blue for comparison. Both spectra share the same overall shape suggesting that similar luminescent centers are activated.

After the initial forming, a confocal PL scan is performed on the device (see Fig. S8b). A bright spot is observed in the gap, hinting the presence of new emission centers–generated after the reverse-bias switching. To better distinguish the emission from these newly generated emission centers in the gap, a 50 nm bandpass optical filter centered at 850 nm is placed before the APD. The confocal scan with filter is shown in Fig. S8d.

The PL spectra of both the gap and SiO_x cladding are shown in Fig. S8c. The spectrum of the gap (shown in red) features a shoulder in the spectral range of 700 nm to 900 nm confirming the presence of electrically activated luminescence species. Based on these PL measurements, one can see that the emission centers generated during reverse-bias switching are similar to the ones observed after forward-bias switching reported in Fig. 5.

The *I* - *V* sweep cycles of the device during reverse-bias switching are shown in Fig. S9. In total 24 *I* - *V* cycles have been applied to the very device. Note that cycle 1 is the electroforming cycle, and the electroforming (dielectric breakdown) voltage is ~ -23 V. The applied voltages are negative as we apply a negative voltage to the Ag electrode and keep the Pt electrode at ground potential. The reverse-bias EL spectrum in Fig. S8a corresponds to the 24th *I* – *V* cycle shown in Fig. S9b.

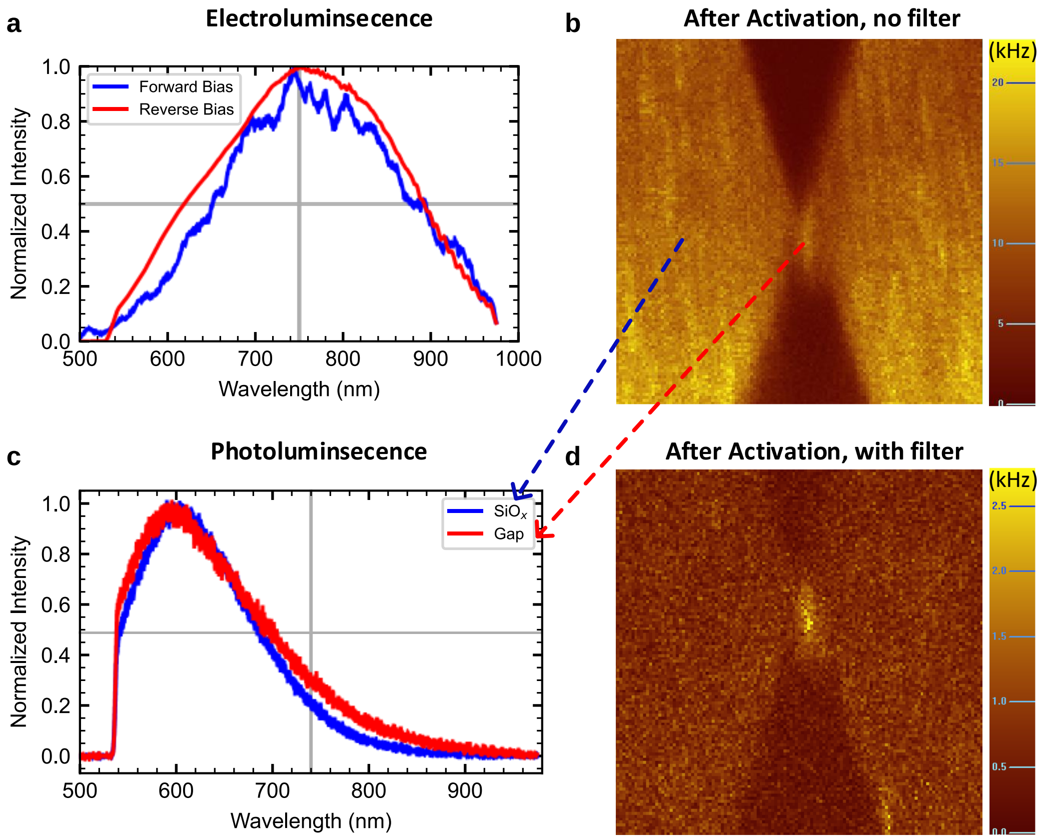

Fig. S8 | Investigation of luminescence under forward- and reverse-bias. Before reverse-bias switching, no forward-bias switching has ever been applied, and no Ag has migrated into the gap. **a**, The measured EL spectrum of a device under reverse-bias switching (red curve). For comparison, the EL spectrum of another device under forward-bias switching is plotted in blue. The EL spectra of both the forward- and reverse-bias switching are similar. Please note that the emission spectra have been normalized. In reality, the reverse-biased spectrum is more intense. **b**, Confocal PL scan of one device after reverse-bias switching. The PL in the gap after reverse-bias switching is weaker than one would see under forward-bias switching. Nevertheless, a luminescent spot is still visible in the gap. The laser power is 300 nW with an integration time of 0.5 ms per pixel. **c**, The measured PL spectra in the gap and in the SiO_x cladding outside of the gap under reverse-bias switching. The PL spectrum of the gap indicates a shoulder response centered around 750 nm, which is also observed in the PL scan taken under forward-bias switching, as shown in Fig. 5d. **d**, Confocal PL scan of one device after reverse-bias switching, with a 50nm optical bandpass filter centered at 850 nm, the laser power is also 300 nW with 0.5 ms per pixel integration time, same as Fig. S8b. This confirms that newly generated species with luminescence at a wavelength around 850 nm are present, similar to what one would expect in the forward-bias case in Fig. 6d of the main text.

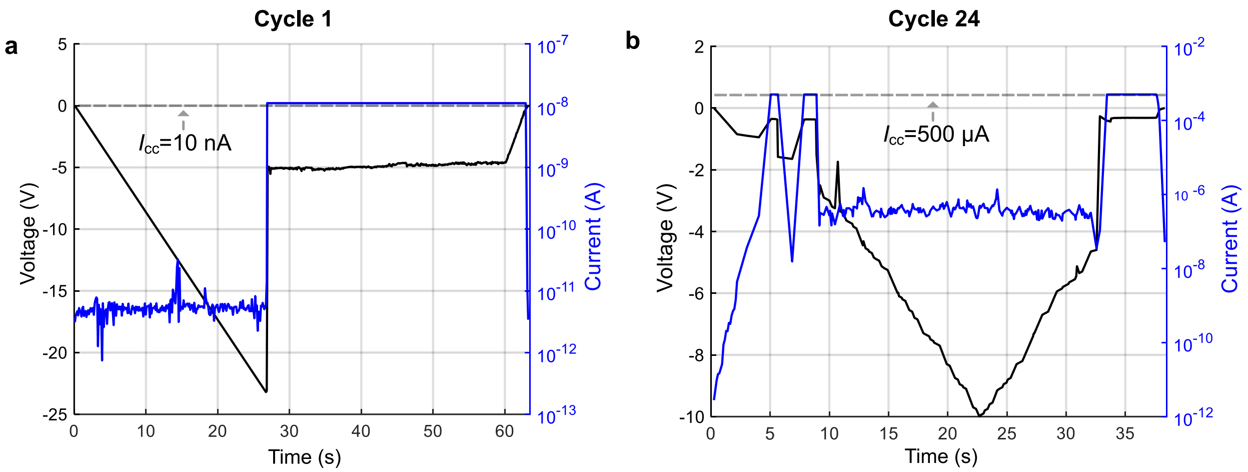

Fig. S9 | *I* - *V* curve of the reverse-bias voltage sweeps of the APS. The applied voltage (left axis) and measured current (right axis) are plotted. **a**, The first reverse-bias sweep cycle, the first electroforming (dielectric breakdown) occurs at ~-23 V. The compliance current is 10 nA. **b**, The 24th reverse-bias sweep cycle. The corresponding EL spectrum is recorded and shown in **Fig. S8**a. The compliance current is 500 µA.

VIII. PL lifetime measurement

PL lifetime measurements are performed to determine the lifetime of the luminescent species at play in our investigations.

The PL lifetime measurement setup is depicted in Fig. S1b. To measure the PL lifetime, the diode laser is operated in pulsed mode delivering 85 ps pulses at a 20 MHz repetition rate. The APD is connected to a TCSPC board in order to build a histogram of the PL photon arrival time with respect to the excitation pulses.

A confocal scan on a device after performing a few cycles of resistive switching is shown in Fig. S10a. To differentiate the switching-induced PL peak around 875 nm, a 50 nm bandpass filter centered at 850 nm is again placed before the APD. The corresponding confocal scan with the filter is shown in Fig. S10b. As already observed in other devices, the filtered PL confocal scan shows substantially stronger PL in the gap compared to the SiO_x background, clearly confirming that new light-emitting species are created.

The PL lifetimes are acquired by performing time-correlated single-photon measurements for the SiO_x cladding obtained without a bandpass filter and in the gap taken with the filter. The results are shown in Fig. S10c and Fig. S10d, respectively. The green line in Fig. S10c is the instrument response function (IRF) obtained by sending an attenuated laser beam directly to the APD. The blue curve is the decay histogram of the background PL recorded from the SiO_x cladding. The dashed black curve is a fit to the data using a double-exponential decay of the form $I = A_1 e^{-\tau_1} + A_2 e^{-\tau_2}$, indicative of the presence of the decaying populations within the probed area. A_1 and A_2 are the respective weights of the two components, and τ_1 and τ_2 are their respective decay times. The fit takes into account a deconvolution algorithm of the IRF to estimate this set of four parameters. The same procedure is applied to characterize the decay dynamics of the electrically-induced defects located in the gap shown in Fig. S10d. The extracted parameters for both curves are summarized in Table S2. The unfiltered SiO_x luminescence features a dominant fast decay component at 0.16 ns and a smaller contribution with longer excited-state dynamics (~ 2 ns). We cannot distinguish if the presence of a double exponential dynamics stems from two different subpopulations of the luminescence defects, relaxation from separated energy levels within the same family of defects, or spatially different environments affecting the photophysics of the centers. For the newly created PL, the characteristic time of the slower dynamics is drastically reduced to about 8 ns. The slow decay time in the

gap is 10 times longer over the lifetime of Si nanocore reported at a given wavelength[4, 9]. The relative contributions of the two decay components are almost equilibrated in this measurement.

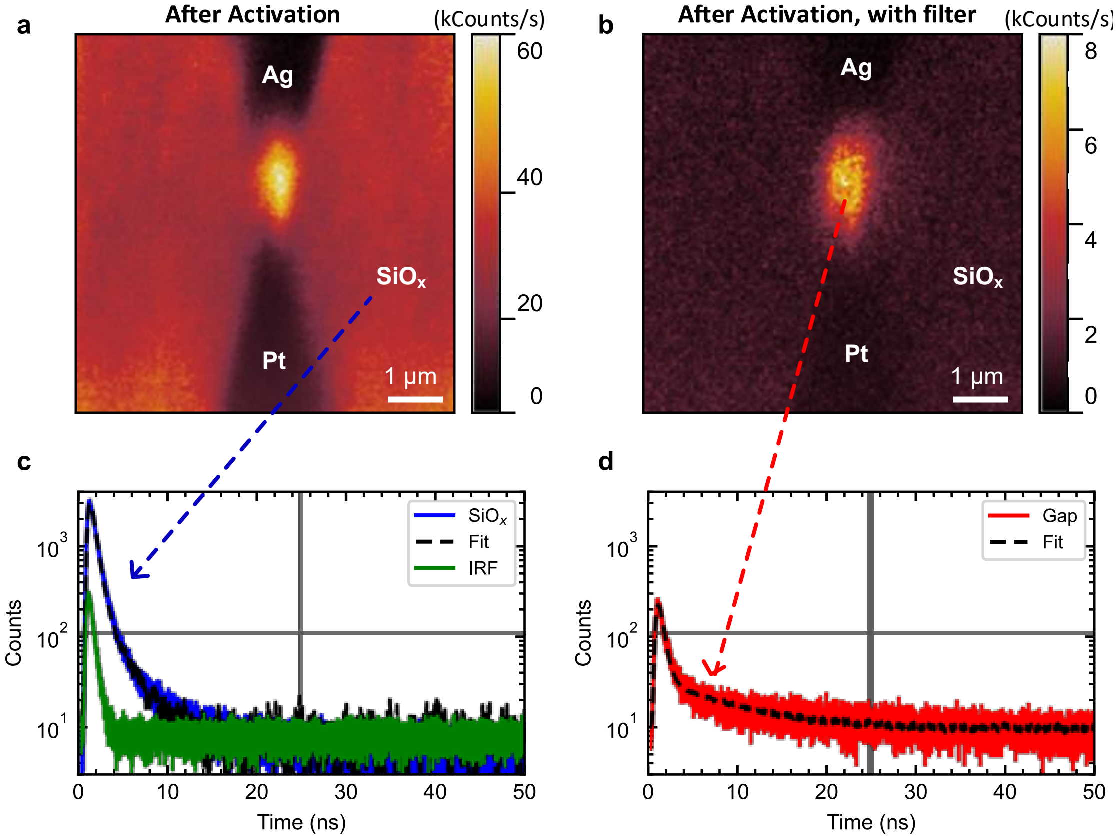

Fig. S10 | PL lifetime measurement. a, Confocal PL scan of one device after resistive switching. **b**, Confocal PL scan shot with a 50 nm bandpass filter centered at 850 nm. The emission centered at 875 nm is now clearly visible. **c**, PL lifetime of the deposited SiO_x cladding without a filter. **d**, PL lifetime of the emission in the gap, with filter.

Table S2 | Lifetime fitting parameters.

	A_1	τ_1	A_2	τ_2
SiO_x	0.81	0.16 ns	0.16	1.94 ns
Gap	0.55	0.19 ns	0.45	7.93 ns

IX. EL and PL spectrum differences

The non-perfect overlapping between the EL and PL spectra can be understood from a series of combined arguments.

In case of EL, electron injection in the emissive center is required to populate an excited state that eventually will relax radiatively. This injection process requires (i) an electron with enough energy to pump the metastable level and (ii) the immediate proximity of the defects to the electron trajectory to enable tunneling and trapping. Here, the excitation energy provided by the applied bias is up to 5 eV. Considering that the detected EL peaks at roughly 1.6 eV, an electron injected at maximum energy available must decay through a series of non-radiative channels before reaching a radiative energy level. For PL excitation, the maximum energy is 2.4 eV. Non-radiative relaxation pathways are also required to bring the photo-excited electron down to a metastable level, but the lowest excited state reached is not necessarily at the same energy for electron injection and optical pumping.

The number of emissive centers that can be excited during the acquisition of a spectrum is obviously different between the two excitation mechanisms. For PL, all emissive defects with a non-zero projection of their dipole absorption moment aligned with the incident polarization and present within the confocal excitation area may contribute to PL spectra. The response is thus a superposition of all these centers and the shape of the PL spectrum reflects the inhomogeneous spectral distribution weighted by the efficiency of all contributors. This is vastly different for EL since only defects connected to the current path and filament forming may be excited and there is no evidence that a given defects can be excited by the two excitation mechanisms.

X. Differences to inelastic electron tunneling

There are several properties of the APS that are hardly explained by inelastic tunneling.

First, we note the consistent observation of a correlation between light emission and large current changes as discussed in Fig. 3b of the main manuscript. Emission is systematically observed concomitant to discrete current jumps. To make it clearer, we plot in the graph below (Fig. S11) the mean photon yield and the total integrated current change for each pulse recorded during the time trace of Fig. 3. The mean photon yield is calculated by dividing the mean photon count by the mean current within a pulse. The total integrated current change value is $(R\text{-}1)^2$, where R is the ratio of current variation defined in the main text. This figure unambiguously demonstrates the relationship between the photon activity. We also note that the photon rate in Fig. 3b is not linked to the number of charges transported in the gap. As light emission from inelastic tunneling is directly related to electron flow it is unlikely to explain light emission only observed during discrete transients and changing currents.

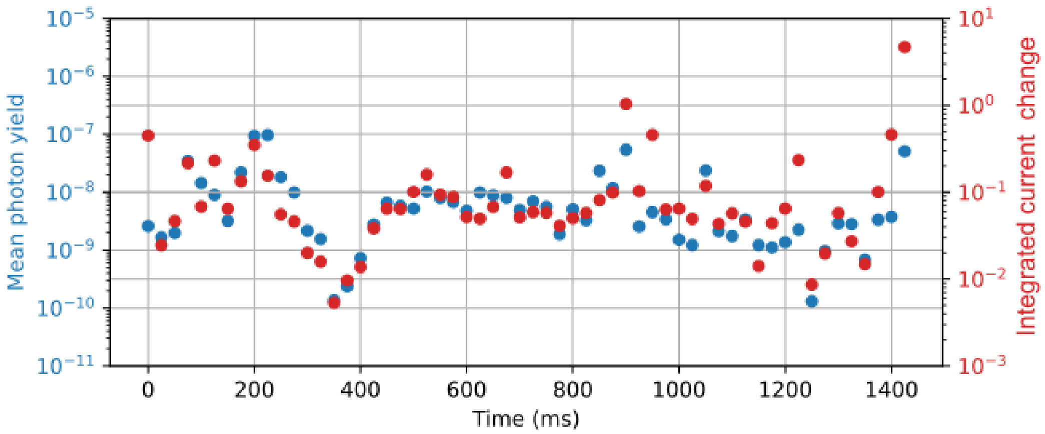

Fig. S11 | Mean photon yield (left axis) and integrated current change (right axis).

Another argument helping to rule out the inelastic tunneling as a main mechanism for light emission is that we never observed the quantum cutoff. The quantum cutoff is the hallmark of inelastic tunneling light emission. The applied bias sets the highest photon energy that can be detected. For Fig. 3d this is 5 eV, corresponding to a cut-off wavelength of 248 nm. If inelastic electron tunneling would be at play, we would therefore expect to observe higher order resonances of the antenna and/or filament in the short wavelength range. In all our acquired spectra we did not record light emission below a wavelength of 500 nm.

References

1. Novotny, L. & Hecht, B. Principles of nano-optics., 1-564 (2009).
2. Parzefall, M. & Novotny, L. Optical antennas driven by quantum tunneling: a key issues review. *Reports on Progress in Physics* **82**, 112401 (2019).
3. Agio, M. Optical antennas as nanoscale resonators. *Nanoscale* **4**, 692-706 (2012).
4. Kanemitsu Y. Luminescence properties of nanometer-sized Si crystallites: core and surface states. *Physical Review B* **49**, 16845-16848 (1994).
5. Ni, Z. Y. et al. Silicon nanocrystals: unfading silicon materials for optoelectronics. *Materials Science and Engineering: R: Reports* **138**, 85-117 (2019).
6. Anger, P., Bharadwaj, P. & Novotny, L. Enhancement and quenching of single-molecule fluorescence. *Physical review letters* **96**, 113002 (2006).
7. Bharadwaj, P., Novotny, L. Spectral dependence of single molecule fluorescence enhancement. *Optics Express* **15**, 14266 (2007).
8. Tsuruoka, T. *et al*. Forming and switching mechanisms of a cation-migration-based oxide resistive memory. *Nanotechnology* **21**, 425205 (2010).
9. Roy, S. *et al*. Fluorescence lifetime analysis and fluorescence correlation spectroscopy elucidate the internal architecture of fluorescent silica nanoparticles. *Langmuir* **26**, 13741-13746 (2010).
